# Cancer développé sur dilatation kystique de la voie biliaire: à propos d’un cas

**DOI:** 10.11604/pamj.2014.19.346.5110

**Published:** 2014-12-03

**Authors:** Mehdi Soufi, Mohammed Khalid Lahlou, Bouziane Chad

**Affiliations:** 1 Division of Digestive Surgery, Oujda CHU, Faculty of Medicine, University Mohammed First, Oujda, Maroc; 2 Service de Chirurgie Viscérale B, CHU Avicenne, Rabat, Maroc

**Keywords:** Kyste du cholédoque, voie biliaire, dégénérescence, chirurgie, radiothérapie, Choledochal cyst, bile duct, degeneration, surgery, radiotherapy

## Abstract

Les auteurs rapportent un cas de dilatation kystique du cholédoque intra- pancréatique découvert chez une femme de 46 ans et compliqué d'un carcinome tubulo-papillaire n'envahissant pas le pancréas. Le traitement a consisté en une duodénopancréatectomie céphalique avec un curage ganglionnaire et anastomose hépatico-jéjunale; la survie était de 30 mois; les auteurs abordent les aspects radiologiques, anatomopathologiques, thérapeutiques et pronostiques de ces cancers développé sur dilatation kystique de la voie biliaire et discutent la place d'une radiothérapie complémentaire de la chirurgie susceptible d'améliorer la survie.

## Introduction

La dilatation congénitale de la voie biliaire principale, plus couramment dénommée dilatation kystique du cholédoque est une malformation rare décrite surtout au Japon [1] et dans les autres pays d´Asie alors que les séries européennes ou américaines ne comptent dans leur ensemble que quelques dizaines d´observations. Cette affection touche le plus souvent le jeune enfant. Les formes de l´adulte correspondent à des lésions présentes déjà dans l´enfance mais passées inaperçues ou tolérées. A peu prés 3.000 cas ont été rapportés dans la littérature mondiale [2]. Depuis les constatations de Irwin et Morrison en 1944, ces lésions sont considérées comme des états précancéreux. Actuellement, il est montré que la prévalence de la dégénérescence augmente avec la durée d´évolution: le risque de cancérisation inférieur à 1% avant l´âge de 10 ans, ce risque dépasse 15% après l´âge de 20ans [3, 4]. Nous voudrions à l´occasion d´une observation qui nous paraît démonstrative insister sur le risque de cancer dans les formes découvertes à l´âge adulte, et sur l´importance de l´imagerie médicale pré-opératoire assurant une bonne approche diagnostique de la malignité et permettant de prévoir une attitude chirurgicale agressive dans ce cas.

## Patient et observation

Les auteurs déclarent avoir reçu le consentement écrit de la patiente pour reporter ce cas. M^me^ F.E., âgée de 46 ans, était hospitalisée dans notre unité, en juillet 2002, pour coliques hépatiques datant de 4 mois associées à un ictère avec urines foncées et selles décolorées spontanément résolutif. L´interrogatoire relevait la notion d´épisodes ictériques similaires pendant l´adolescence et une cholécystectomie à l´âge de 25 ans, faite en dehors de notre service, au cours de laquelle l´opérateur signalait une dilatation de la voie biliaire principale sans obstacle. Les investigations ayant conduit à cette première intervention n´étaient pas précisées. A l´admission, l´examen de l´abdomen était sans particularité. Le bilan hépatique était strictement normal.

**Figure 1:**
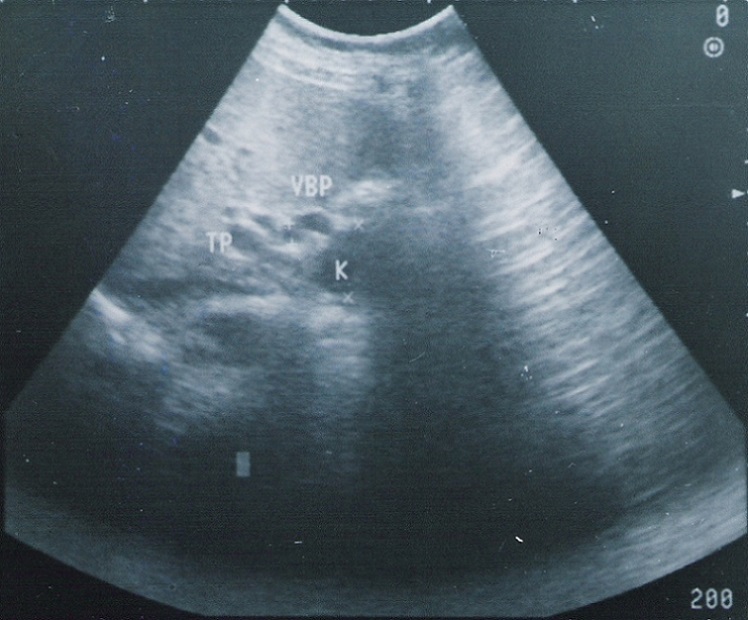
Échographie: image kystique en continuité avec la voie biliaire principale

**Figure 2:**
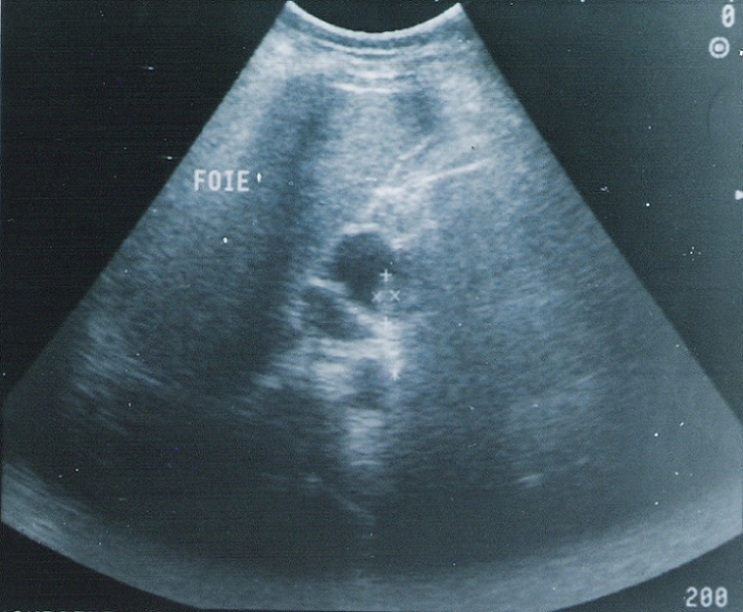
Échographie: tumeur végétante aux dépens de la paroi kystique

**Figure 3:**
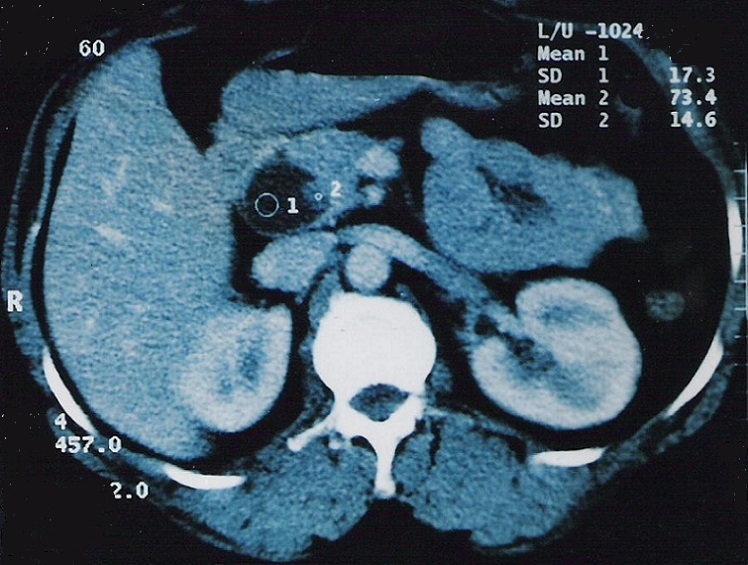
Tomodensitométrie: image kystique intra- pancréatique avec bourgeon pariétal

A l´échographie, il existait une masse de la loge pancréatique semblant se continuer avec la voie biliaire principale, d´échostructure liquidienne, mesurant 29 sur 36 mm et contenant un petit bourgeon échogène de 6 sur 16 mm (Figure 1, Figure 2). Une tomodensitométrie avec injection de produit de contraste montrait une image kystique au sein de la tête du pancréas compatible avec une dilatation kystique de l´extrémité distale de la voie biliaire et qui comportait au niveau de sa paroi une formation tissulaire (Figure 3). Le diagnostic présumé était celui de cancer survenu sur dilatation kystique du cholédoque. L´exploration chirurgicale n´avait pas décelé de dissémination hépatique ou péritonéale. Le pancréas était d´aspect normal. A la cholangiographie per-opératoire, la voie biliaire principale était dilatée sur toute sa longueur et tout particulièrement au niveau du bas cholédoque; il existait une légère dilatation de la convergence biliaire sans dilatation kystique des voies biliaires intra-hépatiques; le passage duodénal du produit de contraste était satisfaisant. Une duodéno-pancréatectomie céphalique selon Whipple était alors pratiquée en même temps qu´une résection de la presque totalité de la voie biliaire extra-hépatique. L'examen extemporané de la tranche biliaire était indemne.

**Figure 4:**
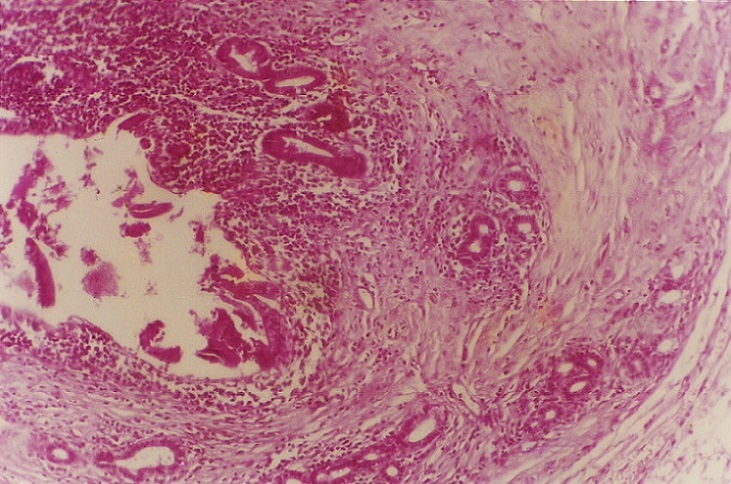
Histologie (grossissement x 4) paroi cholédocienne avec en intra- luminal une prolifération tumorale tubulo-papillaire

**Figure 5:**
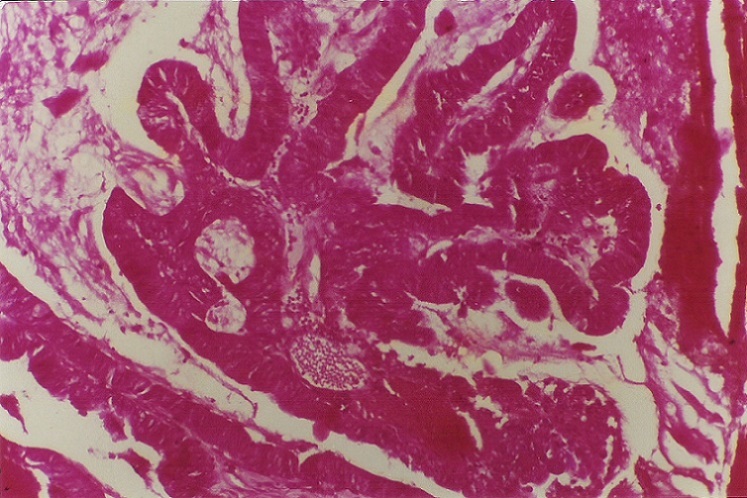
Histologie (grossissement x 40) prolifération tumorale tubulo-papillaire intra- luminale

Sur une anse montée en Y, l´anastomose bilio-digestive intéressait la convergence biliaire. La tranche de section pancréatique, au niveau de laquelle le wirsung n´était pas dilaté, était anastomosée en termino-latéral à l´anse jéjunale montée. Les suites post-opératoires immédiates étaient simples. L´étude anatomo-pathologique de la paroi de la voie biliaire montrait des lésions ulcératives avec hyperplasie épithéliale; des zones de dysplasie modérée et sévère étaient présentes au niveau de la partie dilatée intra-pancréatique. La paroi cholédocienne était siège d´un adénocarcinome tubulo-papillaire bien différencié du type excréto-biliaire, à développement strictement intra-luminal n´infiltrant pas le parenchyme pancréatique (Figure 4, Figure 5). L´extrémité supérieure de la pièce ne comportait aucune dysplasie. Les prélèvements biopsiques au niveau du foie et des ganglions pédiculaires et rétro-duodéno-pancréatiques étaient négatifs. Après l´intervention, l´état de la patiente était satisfaisant. Aucune irradiation ni chimiothérapie complémentaires n´étaient administrées. Une récidive d´ictère après 28 mois d´évolution était en rapport avec des métastases hépatiques. La malade devait décéder deux mois plus tard.

## Discussion

**Figure 6:**
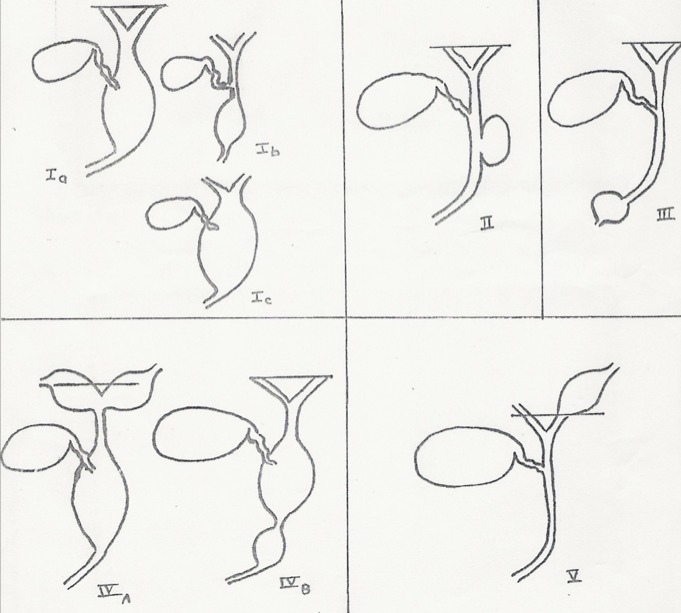
Classification des dilatations kystiques congénitales de la voie biliaire principale selon Todani

La dilatation kystique de la voie biliaire (DKVB) peut se présenter sous divers aspects morphologiques [1, 2]. La classification d´Alonso-lej, la plus souvent citée, date de 1959 et distingue 4 types [3]. Plus tard, Todani a proposé une classification modifiée, actuellement plus largement employée car tient compte de l´état des voies biliaires intra-hépatiques [4, 5] (Figure 6). Cette classification de est utile pour l´analyse de la pathophysiologie et de la détermination de la stratégie thérapeutique [6]. De 1990 à 2006, nous avons eu à traiter 9 cas de DKVB dans notre unité chirurgicale de l´adulte. Cette affection est rare dans notre pays; à notre connaissance une quarantaine de cas ont été décrits dans nos différents centres. Dans notre série, 7 cas répondaient au type I de la classification de Todani; dans la littérature cette forme est prépondérante (80% des cas). Un de nos malades présentait une dégénérescence de la voie biliaire. L´analyse des constatations radiologiques et per-opératoires permet de le considérer comme type Ib car la dilatation kystique n´intéressait qu´un segment de la voie biliaire extra-hépatique alors que les voies biliaires intra-hépatiques ne participaient pas au processus.

Actuellement, il est bien établi que la DKVB est la conséquence d´une anomalie de la jonction bilio-pancréatique. Il s´agit d´une disposition anatomique anormale de la confluence bilio- pancréatique sous la forme d´un canal commun (CCBP) long de 10 mm et plus [7, 8]. Cette anomalie est retrouvée dans 70% des cas dans le type Ia et dans 30% des cas dans le type Ic [9]. Le CCBP favorise le reflux de liquide pancréatique dans les voies biliaires comme en témoigne d´ailleurs le dosage de l´amylase dans la dilatation kystique et dans la vésicule biliaire. Ce reflux provoque des altérations histologiques de la paroi du canal biliaire et peut expliquer par ce biais non seulement la survenue de la DKVB mais aussi sa transformation maligne [3, 10]. En effet, la régénération continue de l´épithélium biliaire dénudé évolue au long court vers la métaplasie intestinale puis vers la dysplasie. Des études anatomo-pathologiques ont pu mettre en évidence des lésions hyperplasiques, dysplasiques, et carcinomes in situ disséminés illustrant l´hypothèse d´un continuum évolutif vers un stade invasif [11, 12]. Le cas de dégénérescence que nous rapportons pourrait bien être inclus dans ce cadre là; des zones de dysplasie modérées et sévères, à différents sites de la dilatation, étaient associés à une lésion tumorale qui du reste ne dépassait pas la musculeuse. Todani, dans une série de plus de 300 cas de cancers des voies biliaires survenus sur DKVB et/ou anomalie de la jonction bilio-pancréatique, a trouvé la répartition suivante: 50% des cancers au niveau de la voie biliaire extra-hépatique, 46,5% dans la vésicule biliaire, 2,3% dans les voies biliaires intra-hépatiques, 0,7% dans le foie et 0,7% au niveau du pancréas [13].

Surtout observées au Japon, les formes dégénérées de DKVB se rencontrent le plus souvent chez la femme [14] et le sexe ratio F/H voisin de 2,5 est identique à celui des DKVB; le cancer de la voie biliaire principale étant un peu plus fréquent chez l´homme. Les formes dégénérées de DKVB ne s´observent pratiquement que chez l´adulte dans la troisième décade [3, 4, 7, 14] alors que l´âge moyen de survenue de cancer de la voie biliaire est voisin de 70 ans. La dégénérescence est située habituellement dans la zone dilatée où peut stagner le suc pancréatique. Cette dégénérescence siège dans la partie pédiculaire de la voie biliaire dans le type I et peut intéresser la vésicule biliaire dans le type Ic [13] ou les voies biliaires intra-hépatiques dans le type IVA [15]. La simple dérivation interne kysto-duodénale ou kysto-jéjunale majore le risque de transformation maligne [16]. En effet le reflux de liquide intestinal dans la voie biliaire contribue à l´accélération des phénomènes d´ulcération, régénération épithéliale métaplasique et par conséquent l´évolution vers le cancer [14]. Dans la littérature, des cancers sont apparus après dérivation kysto-duodénale dans un délai de 2ans [3, 7]. L´adénocarcinome est le type histologique le plus fréquent (90% des cas); des formes mixtes avec des zones d´hépithéliomas malpighiens ne sont pas rares [2]. Le type papillaire aurait le meilleur pronostic [12]. Sur le plan clinique, aucun signe d´appel n´est particulier au cancer développé sur DKVB [3]. La tumeur siégeant dans la zone dilatée ne devient que tardivement obstructive [3] et lorsque l´ictère retentionnel apparaît, la lésion est déjà très évoluée. L´ictère récidivant après dérivation kysto- digestive antérieure doit toujours susciter des inquiétudes. L´amaigrissement, une masse fixe, une hépatomégalie témoignent souvent d´une extension dépassée.

Le diagnostic radiologique des DKVB, chez l´adulte, ne devrait pas poser de difficulté dans la majorité des cas [16]. Seules les formes intéressant la partie basse de la voie biliaire principale risquent de poser un problème de diagnostic différentiel avec un pseudo-kyste ou une tumeur kystique du pancréas [17] comme c´était le cas de notre patiente ou même un kyste hydatique fréquent sous nos latitudes. A l´échographie, la nature liquidienne de la lésion, son siège épiportal et sa continuité avec la voie biliaire éliminent un exceptionnel anévrisme veineux du confluent mésentérico-porte [17]. Si la lésion est de grande taille, cet examen semble plus intéressant que la tomodensitométrie pour déterminer l´origine de la masse en raison de la multiplicité des plans de coupes possibles. Le diagnostic pré-opératoire de la malignité est essentiel pour une bonne approche chirurgicale. La présence d´images échogènes intra-kystiques peut être en rapport avec des calculs un sludge ou un cancer [1, 10]. La tomodensitométrie avec injection de produit de contraste permet dans ce cas de préciser la nature tissulaire d´un nodule pariétal et de détecter les toutes petites lésions [18]. La cholangio-pancréatographie per-endoscopique et/ou la cholangio-graphie transhépatique permettent de bien étudier l´anatomie des voies biliaires en particuliers intra-hépatiques et de préciser les deux extrémités de la dilatation et sa connexion avec le cholédoque sain [1, 4, 5, 9]. Toute irrégularité ou sténose de la voie biliaire doit suggérer la malignité [1, 18]. Le diagnostic peut être renforcé par l´examen cytologique de la bile obtenu au cours de ces deux procédures de cholangiographie [7, 14]. Récemment la cholangio-IRM a été mise à profit dans l´étude morphologique des dilatations kystiques est devenue l'examen de référence [19].

L´exploration radiologique per-opératoire est surtout utile quand les données de l´exploration pré-opératoires sont insuffisantes [2]. Dans le cas que nous rapportons, l´injection du produit de contraste en per-opératoire ne faisait que dilater la partie kystique avec une faible opacification des voies biliaires intra-hépatiques et de la jonction bilio-pancréatique. Du point de vue chirurgical, deux éléments sont déterminants pour la prise en charge de ces formes dégénérées: l´association de dysplasie multifocale au cancer et la présence toujours possible des foyers carcinomateux à différents endroits de la voie biliaire [1, 12, 19]; la nécessité de rétablir la continuité digestive à un niveau où l´épithélium biliaire n´est ni ulcéreux ni fibreux afin d´éviter les fistules et les sténoses anastomotiques [20]. En plus de l´exérèse de la totalité de la poche kystique et de la vésicule biliaire, il est donc impératif de remonter vers le haut sur le canal hépatique voire au niveau de la convergence [1, 2, 3, 8]. Une anastomose hépatico-jéjunale est actuellement la plus préférée et ce quel que soit le type de dilatation kystique. La résection de la convergence doit être d´autant plus étendue que celle-ci est kystique. Lorsqu´il existe des anomalies intra-hépatiques unilobaires associées, une résection hépatique partielle doit être envisagée [8, 15, 16].

En cas de dégénérescence du cholédoque intra-pancréatique ou si le pancréas est envahi [2], une duodéno-pancréatectomie céphalique est le geste adéquat [7]. Le curage ganglionnaire est associé à la demande guidé au besoin par l´examen histologique extemporané. Ce curage porte sur la chaîne hépatique, coeliaque, rétro-duodéno-pancréatique et lombo-aortique [1]. Une attitude chirurgicale agressive d´exérèse loco-régionale étendue doit être tentée en l´absence de dissémination à distance d´autant qu´il s´agit de sujets jeunes [21]. Le bénéfice de ce type de chirurgie reste à démontrer car le pronostic est généralement mauvais et la survie dépasse rarement deux ans [22]. Le pronostic est plus sombre chez les malades ayant eu une kysto-dérivation sans résection kystique. Dans une série de Todani comptant 105 exérèses avec 76 exérèses de la poche kystique et de la vésicule biliaire et 29 exérèses duodéno-pancréatiques, il n´y avait aucun survivant à 5 ans [2, 12]. En ce qui concerne notre malade, la survie était de 20 mois malgré l´aspect peu infiltrant de la tumeur et son caractère réséquable. Dans une optique curative et en raison des résultats médiocres de la chirurgie, la radiothérapie adjuvante dirigée sur les reliquats tumoraux microscopiques peut être justifiée [12]. En effet, dans certaines séries, l´irradiation post-opératoire, a permis d´obtenir un allongement de survie dans les carcinomes des voies biliaires extra-hépatiques. La tolérance de l´anastomose hépatico-jéjunale à cette radiothérapie est bonne pour des doses ne dépassant pas 55 GY [12].

## Conclusion

Le cancer développé sur dilatation kystique de la voie biliaire est de pronostic sombre. Le traitement préventif par la résection radicale de cette malformation doit se faire dans l'enfance. Chez l'adulte, l'examen histologique extemporané de toute la paroi kystique doit être systématique au cours de la résection.
